# Primary parotid tuberculosis mimicking parotid neoplasm: a case report

**DOI:** 10.1186/1752-1947-2-62

**Published:** 2008-02-26

**Authors:** Hakan Birkent, Serdar Karahatay, Timur Akcam, Abdullah Durmaz, Onder Ongoru

**Affiliations:** 1 Department of Otolaryngology-Head&Neck Surgery, Gulhane Military Medical School, Ankara, Turkey; 2 Department of Pathology, Gulhane Military Medical School, Ankara, Turkey

## Abstract

**Introduction:**

Tuberculosis of the parotid gland is a rare clinical entity which causes some difficulties in diagnosis because of the similarities in presentation to that of a neoplasm. Diagnosis mainly relies in the treating physician having a high index of suspicion. The diagnosis is generally overlooked by otolaryngologists and most cases are undergoing unnecessary surgery.

**Case presentation:**

A 20-year-old male presented with a mass in the right parotid region. The mass had been present for one year. Physical examination revealed a mobile, non-tender mass occupying the superficial lobe of the right parotid gland. Radiologic investigations revealed a well-defined, solid, mass lesion located in the posterior part of the superficial lobe of the right parotid gland. A provisional diagnosis of a neoplasm of the parotid gland was made and a right superficial parotidectomy was performed. Histopathologic examination of the specimen was reported as tuberculosis of the parotid gland. The patient was commenced on antitubercular chemotherapy.

**Conclusion:**

Although rare, tuberculosis should be kept in mind and considered in the differential diagnosis of patients presenting with a solitary tumor in the parotid gland in order to avoid unnecessary surgery.

## Introduction

Tuberculosis is a necrotizing granulomatous disease with varied clinical presentations and a wide distribution. The lungs are most commonly involved. Extrathoracic forms of the disease account for approximately 20% of overall active tuberculosis and can be seen in the kidneys, bones, meninges, and lymph nodes [1,2]. Tuberculosis lymphadenitis is the most common extrathoracic form and the cervical lymph nodes, including lymph nodes in and around the salivary glands, are the ones most frequently involved [2].

However, parotid gland involvement is extremely rare, even in countries in which tuberculosis is endemic [3]. Less than 200 cases have been reported since the first description of this condition by von Stubenrauch in 1894 [4,5]. Clinically, it generally presents as a slow growing mass indistinguishable from a malignancy [6]. The diagnosis of parotid tuberculosis needs a high degree of clinical suspicion. If there is no history of pulmonary tuberculosis and no relevant symptoms, diagnosis can be extremely difficult. Therefore it is generally overlooked by otolaryngologists and most cases are undergoing unnecessary surgery.

We herein report a case of parotid tuberculosis presenting as a parotid mass mimicking a neoplasm and discuss the clinical features, diagnostic methods, histopathologic findings, and treatment of this rare clinical entity.

## Case presentation

A 20-year-old male presented with a mass in the right parotid region of one year duration. It was enlarging gradually and was not associated with any other symptoms. His medical history was nonrevealing for any systemic disease. He gave no personal or family history of tuberculosis, and no other relevant symptoms such as night sweats, weight loss or pulmonary symptoms. Physical examination revealed a mobile, non-tender mass occupying the superficial lobe of the right parotid gland. There was no overlying skin changes suggestive of parotitis. Facial nerve function was normal. Other physical findings were also normal and there was no palpable cervical lymphadenopathy.

**Figure 1:**
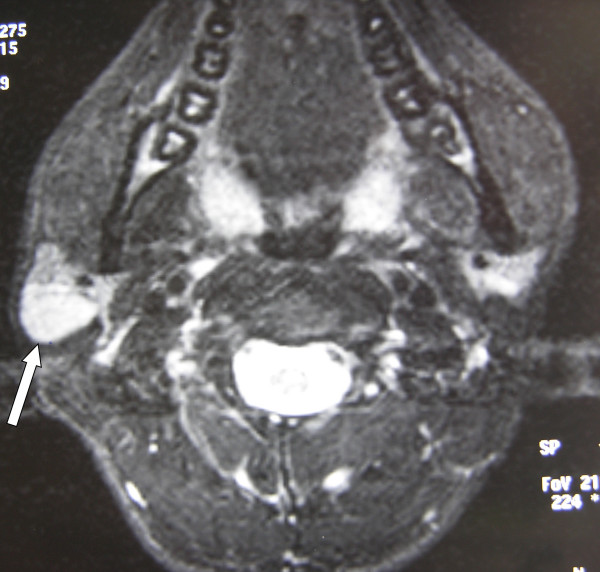
An axial fat saturated T2-weighted image through the parotid gland demonstrates a well-defined high signal intensity mass in the superficial area of the right parotid gland.

A provisional diagnosis of a neoplasm of the parotid gland was made and the patient was investigated accordingly. The complete blood count, erythrocyte sedimentation rate, other biochemical investigations and chest X-ray were normal. Ultrasound examination (US) showed a well-defined, hypoechoic solid mass lesion in the superficial lobe of the right parotid gland. Fine needle aspiration cytology was performed, but the cytological findings were not diagnostic. Magnetic resonance imaging (MRI) revealed a well-defined mass lesion, measuring 16 × 21 × 30 mm in size, hypointense on T1-weighted images, and hyperintense on T2-weighted images with homogenous contrast enhancement, located in the posterior part of the superficial lobe of the right parotid gland (Figure 1).

**Figure 2:**
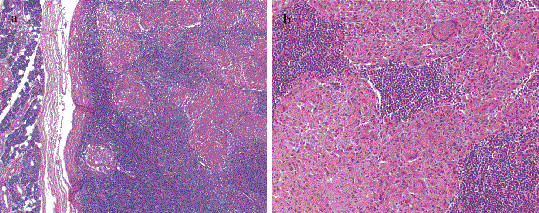
(a) Granulomatous inflammation in a lymph node abutting the parotid gland (H&E ×50), (b) Langhans type giant cells in granulomatous inflammation (H&E ×200).

Because the findings were compatible with a parotid neoplasm, a right superficial parotidectomy was performed. The superficial lobe was removed in toto with the mass while preserving the facial nerve. The postoperative course was uneventful. The histopathologic examination of the specimen was reported as caseating granulomatous infection with Langhans giant cells (Figure 2a,b). An intradermal test with purified protein derivative (PPD) was performed and it was strongly positive with 24 mm of duration. Arrangements were made for the patient to receive antitubercular chemotherapy with rifampicin, isoniazid, pyrazinamid and ethambutol for six months of duration.

## Discussion

Tuberculosis is common in developing countries and increasing in developed countries in recent years due to factors such as the development of resistant strains and co-infection with HIV [7]. Despite tuberculosis commonly involving the lungs, extrapulmonary forms are not at all uncommon and account for approximately 20% of overall active tuberculosis, but the salivary glands appear to be rarely infected [1,3,6]. This may be due to the inhibitory effect of saliva on mycobacteria [6].

The pathogenesis of parotid tuberculosis remains unclear [1]. Involvement of the parotid gland and lymph nodes may develop in two ways. First, a focus of mycobacterial infection in the oral cavity liberates the mycobacterium that ascend into the salivary gland via its duct or pass to its associated lymph nodes via lymphatic drainage. The second pathway involves hematogeneous or lymphatic spread from a distant primary lung focus [2].

There are varied clinical presentations of parotid gland involvement with tuberculosis. It most commonly presents as a localized mass, resulting from infection of intracapsular or pericapsular lymph nodes. It may also present as an acute sialadenitis with diffuse glandular enlargement. In this form the involvement is in the parenchyma of the salivary gland. It may also present as a periauricular fistula or as an abscess [8].

Parotid tuberculosis becomes a real diagnostic problem in the absence of clinical disaese in the lung and without any systemic signs and symptoms. Most of these cases present with a slow growing mass, gradually increasing in size over two to six months and these lesions are almost impossible to distinguish from a parotid neoplasm [9]. Physical examination in general is unrewarding. A chest radiograph may be helpful in cases of associated pulmonary tuberculosis. But less than 50% of patients with extrapulmonary tuberculosis exhibit radiologic evidence of pulmonary disease [10]. Our patient did not have chest radiographic evidence of either active or prior pulmonary tuberculosis. The use of tuberculin skin testing can provide valuable information, but requires an initial suspicion. In our case tuberculin testing was performed after the surgical excision and was strongly positive (24 mm).

The definitive diagnosis of tuberculosis depends on the isolation and identification of mycobacteria from a diagnostic specimen [3]. Maynard stated that there were no methods of distinguishing this infection from a parotid gland neoplasm except by histologic examination [11]. Fine needle aspiration cytology (FNAC) is advocated as a useful and reliable technique for the diagnosis of tuberculosis in the parotid gland [1,3,6]. In parotid lesions FNAC has a sensitivity of 81–100% and specifity of 94–100% [9]. Thus, FNAC should be performed first in the evaluation of a parotid mass. But it is not always contributory to a diagnosis in large parotid neoplasms as these are often necrotic [12]. It is also possible to culture the aspirate, but that needs an initial suspicion and may require a long time period to obtain a result.

Because a negative FNAC report does not rule out a malignant neoplasm, it becomes necessary to utilise other diagnostic aids such as imaging and exploration. Imaging studies generally involve ultrasonographic examination, computerized tomography and/or magnetic resonance imaging. But there is no specific sign of tuberculosis in the parotid with any of these imaging techniques. Since tuberculous infection may involve multiple sites in the parotid gland and periparotid region, MRI may better delineate the nature of the disease than CT or US [9]. Incisional biopsy or drainage should not be used as they may result in the development of cutaneous fistulae. Excisional biopsy becomes mandatory when other investigations are non-contributory. In a meta-analysis by Lee and Liu of 49 cases of tuberculous parotitis, FNAC was helpful in only 10 cases and the diagnosis was established by parotidectomy in 34 cases [1]. In our patient, we could not find any evidence of active disaese elsewhere. The diagnostic tools including FNAC, US, and MRI were unrewarding and surgical excision was performed in order to make the diagnosis.

The mainstay of treatment is medical in the form of anti-tubercular chemotherapy for at least six months. If the diagnosis can be obtained by ancillary diagnostic tools, effective antituberculous chemotherapy can lead to resolution of the lesion, avoiding the necessity of surgery.

## Conclusion

Tuberculosis of the parotid gland is a rare clinical entity which presents difficulties in diagnosis because of the similarity of the presentation to that of a neoplasm. Diagnosis mainly relies on the treating physician having a high index of suspicion. Although rare, tuberculosis should be kept in mind and considered in the differential diagnosis of any patient presenting with a solitary tumor in the parotid gland.

## Competing interests

The author(s) declare that they have no competing interests.

## Authors' contributions

All authors contributed to each stage of this work.HB**, **SK**, **TA**, **AD, and OOall have: (1) made substantial contributions to conception and design, or acquisition of data, or analysis and interpretation of data; (2) been involved in drafting and revising the manuscript; and (3) given final approval of the version to be published.

## Consent

Written informed consent was obtained from the patient for publication of this case report and accompanying images. A copy of the written consent is available for review by the Editor-in-Chief of this journal.
